# Ubiquitination modifications as central regulators of metabolic dysfunction in type 2 diabetes mellitus

**DOI:** 10.3389/fendo.2026.1802982

**Published:** 2026-07-09

**Authors:** Wen-tao Wang, Ze-ya Shi, Hai-long Wang, Zhao-yang Chen

**Affiliations:** 1 Laboratory Animal Center, Shanxi Key Laboratory of Experimental Animal Science and Animal Model of Human Disease, Shanxi Medical University, Jinzhong, China; 2 School of Basic Medicine, Shanxi Medical University, Jinzhong, China

**Keywords:** deubiquitinating enzyme, E3 ubiquitin ligase, glycolipid metabolism, insulin resistance, T2DM, therapeutic target, ubiquitination

## Abstract

Type 2 diabetes mellitus (T2DM) is a multifactorial metabolic disorder characterized by insulin resistance, progressive pancreatic β-cell dysfunction, and multi-organ metabolic dysregulation. Among various post-translational modifications, ubiquitination has emerged as a central regulator of cellular homeostasis, with E3 ubiquitin ligases and deubiquitinating enzymes (DUBs) governing the stability and activity of key signaling proteins. This review systematically elucidates the pivotal role of ubiquitination in the pathogenesis of T2DM. We first outline the fundamental principles of the ubiquitin system, followed by an in-depth discussion of its regulatory mechanisms in insulin-sensitive tissues (liver, skeletal muscle, and adipose tissue) and pancreatic β-cells. We then explore the potential and challenges of targeting specific E3 ligases or DUBs as innovative therapeutic strategies for T2DM. Notably, this review provides three novel perspectives. First, we move from generalized description to enzyme-specific resolution, systematically profiling E3 ligases and DUBs with defined pathological functions in T2DM. Second, we adopt a multi-organ integration approach, highlighting the differential and sometimes heterogeneous roles of the same enzyme across distinct tissues. Third, we shift from a mechanism description a translational orientation, critically evaluating the current status of human evidence, identifying knowledge gaps, and discussing emerging strategies including single-cell omics, spatial transcriptomics, proteomics-based ubiquitination mapping, and artificial intelligence-assisted drug discovery. Collectively, our core conclusion is that E3 ligases and DUBs form tissue-specific regulatory networks governing T2DM pathology. Targeting the ubiquitination system offers new precision therapeutic strategies. Future directions should prioritize integrating multi-omics approaches and AI platforms to accelerate translational applications. This review provides a theoretical foundation for understanding the molecular basis of T2DM and facilitates the development of novel therapeutic interventions.

## 1 Introduction

Diabetes mellitus is a metabolic disorder characterized by chronic hyperglycemia and is primarily classified into type 1 diabetes mellitus (T1DM), type 2 diabetes mellitus (T2DM), gestational diabetes mellitus, and other specific types of diabetes (1). T1DM is traditionally referred to as “insulin-dependent diabetes mellitus,” whereas T2DM has been described as “non-insulin-dependent diabetes mellitus” (2). However, T2DM has emerged as a major global public health concern, driven by the complex interplay of genetic, epigenetic, and environmental factors, and is characterized by multiple pathological mechanisms, including insulin resistance (IR), pancreatic β-cell dysfunction, and elevated hepatic glucose production (3). As a multifactorial metabolic disorder, the global prevalence of T2DM continues to increase, imposing a substantial and growing public health burden (4). The pathophysiological core of T2DM lies in a vicious cycle between IR and progressive pancreatic β-cell dysfunction, accompanied by disrupted metabolic homeostasis across multiple organs, such as the liver, adipose tissue, and skeletal muscle (5). According to the International Diabetes Federation Diabetes Atlas (10th edition), approximately 537 million people aged 20–79 years were living with diabetes worldwide in 2021, corresponding to a global prevalence of 10.5%. China accounts for the largest affected population globally, with an estimated 140.9 million adults with diabetes and a reported prevalence of 10.6% (6). The pathogenesis of T2DM is a progressive process in which environmental factors act upon a susceptible genetic background to trigger IR, ultimately overwhelming the compensatory capacity of β-cells. Understanding this dual-core mechanism is crucial for effective disease prevention, early diagnosis, and comprehensive therapeutic intervention.

**Figure 1:**
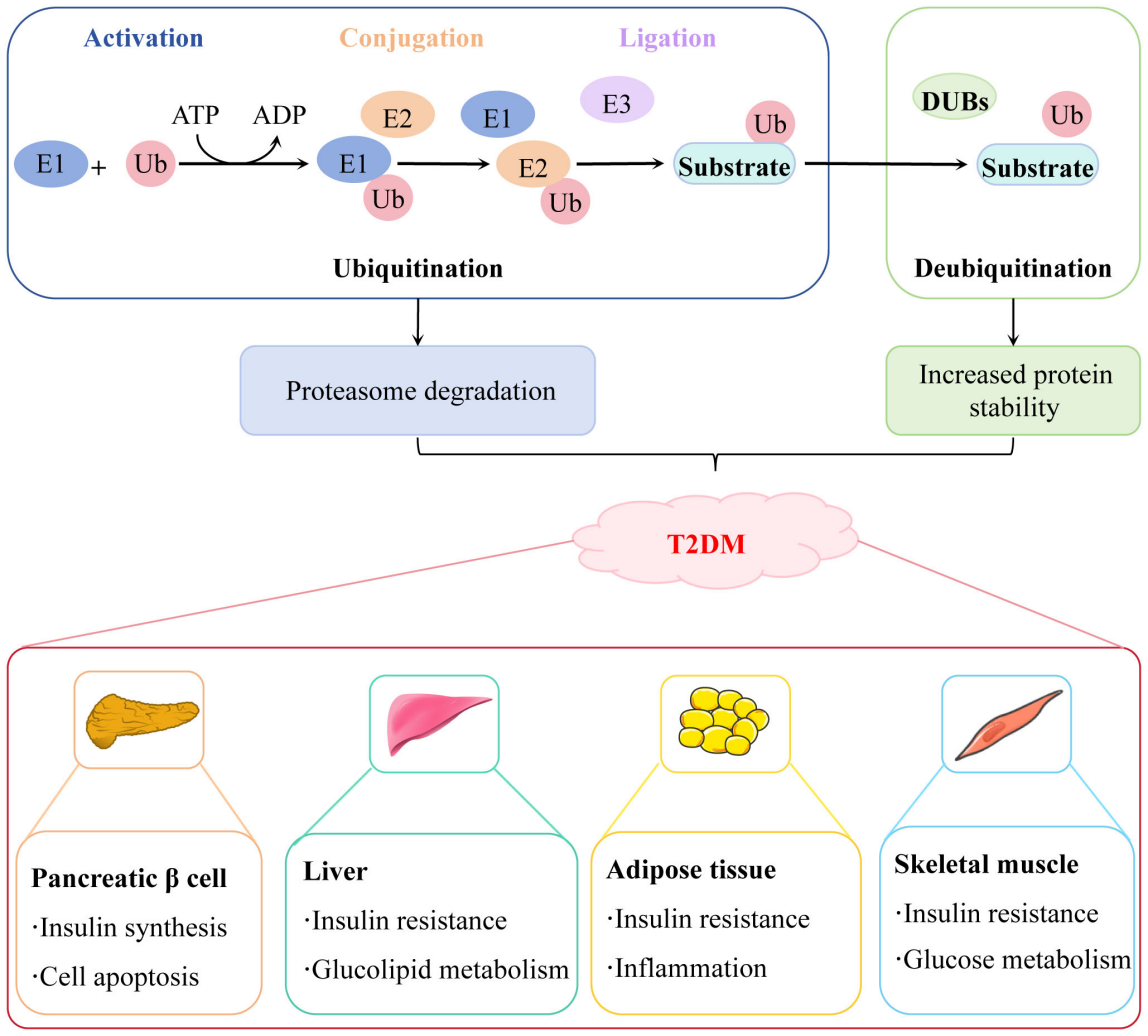
Diagram of the relationship between pathological dysregulation in key target organs and ubiquitination-mediated regulation in T2DM.

Among the various post-translational modification mechanisms, ubiquitination has increasingly been recognized as a central regulator of cellular homeostasis owing to its extensive and precise regulatory functions. This process covalently tags substrate proteins with ubiquitin molecules through a coordinated E1-E2-E3 enzyme cascade reaction, thereby precisely regulating protein stability, activity, subcellular localization, and molecular interactions (7). Ubiquitination exhibits a high degree of complexity and specificity, as distinct ubiquitin chain types and modification sites can activate different signaling pathways, ultimately determining the fate and function of substrate proteins (8). Therefore, ubiquitination plays a pivotal role in cellular metabolism and signal transduction, and accumulating evidence has linked its dysregulation to the pathogenesis of numerous human diseases (9). Notably, ubiquitination modifications permeate nearly all major insulin signaling pathways and metabolic regulatory networks. Even subtle imbalances in ubiquitin-mediated regulation can trigger systemic metabolic dysfunction, providing a crucial molecular basis for metabolic diseases such as T2DM (10). **Figure 1** illustrates the core regulatory network of ubiquitination and deubiquitination across the principal metabolic target organs implicated in T2DM. This dynamic regulatory network is initiated through E3 ubiquitin ligase-mediated substrate recognition and ubiquitin conjugation, processes that are reversed by deubiquitinating enzymes (DUBs), thereby forming a reversible post-translational modification switch. Through this dynamic modulation, ubiquitination precisely regulates the stability, activity, and subcellular localization of key signaling proteins in the liver, skeletal muscle, adipose tissue, and pancreatic β-cells, ultimately influencing their biological functions. Dysregulation of this ubiquitin-centered molecular machinery manifests at the organ level as abnormal hepatic glucose output, impaired glucose uptake in skeletal muscle, chronic inflammation in adipose tissue, and defective insulin secretion by pancreatic β-cells. Collectively, these disturbances constitute the fundamental pathophysiological basis of systemic metabolic dysregulation observed in patients with T2DM.

In recent years, the role of ubiquitination in metabolic diseases has received widespread attention, and several reviews have summarized progress in this field. Xiong et al. (11) employed bibliometric methods to analyze research trends in the ubiquitination field in diabetes, but their focus was on macro-level publication patterns rather than specific molecular mechanisms. Francis et al. (12) focused on the dynamics of ubiquitination during pancreatic β-cell differentiation and dedifferentiation, but did not systematically discuss insulin-target tissues such as the liver and adipose tissue. Zhuo et al. (13), discussed the diabetic immune microenvironment from the perspective of SUMOylation, a modification distinct from ubiquitination. Zhao et al. (14), in a comprehensive review of post-translational modifications in metabolic diseases, covered ubiquitination as one of several modifications, but the limited scope precluded an in-depth discussion of the tissue-specific roles of specific E3 ubiquitin ligases and DUBs. In addition, some reviews have focused on single complications such as non-alcoholic fatty liver disease (15), while others have discussed the interactions between E3 ligases and DUBs or the general mechanisms by which they cooperatively regulate cellular processes (16). None of these reviews have systematically integrated the ubiquitination regulatory network within the core pathological components of T2DM, including IR, β-cell dysfunction, and multi-organ metabolic dysregulation.

This review aims to systematically elucidate the central role of ubiquitination in the pathogenesis of T2DM. We first outline the fundamental principles of the ubiquitin system, followed by an in-depth discussion of its regulatory mechanisms governing insulin signaling sensitivity in major metabolic target tissues, including the liver, skeletal muscle, and adipose tissue. In addition, we provide a detailed analysis of the impact of ubiquitination on insulin secretion, protein quality control, and cell survival in pancreatic β-cells. Finally, we will explore the potential and challenges of targeting specific E3 ubiquitin ligases or DUBs as innovative therapeutic strategies for T2DM. On this basis, this review offers three novel perspectives. First, by shifting from a “generalized description” to an “enzyme-specific resolution,” we systematically profile E3 ligases (such as RNF167, RNF186, and FBXW7) and DUBs (such as USP25 and OTUD5) with defined pathological functions in T2DM, clarifying their substrate specificity, tissue distribution, and upstream/downstream signaling networks. Second, by shifting from a “single tissue” to a “multi-tissue integration” perspective and recognizing T2DM as a multi-organ systemic disease, we systematically integrate the differential roles of E3/DUBs in the liver, adipose tissue, skeletal muscle, and pancreatic islets, with particular emphasis on the functional heterogeneity that the same enzyme may exhibit across different tissues. Third, by shifting from a “mechanistic description” to a “translation orientation,” we not only summarize existing mechanistic evidence but also evaluate the current status of translation from animal models to humans, critically identify knowledge gaps, and prospectively discuss emerging strategies, including single-cell omics, spatial transcriptomics, proteomics-based ubiquitination mapping, and artificial intelligence-assisted drug discovery, with the goal of providing new insights for targeted ubiquitination-based therapies in T2DM. The above discussion is intended to provide a theoretical foundation and future research directions for advancing the understanding of the molecular basis of this disease and for facilitating the development of novel therapeutic interventions.

## 2 Molecular mechanisms of ubiquitination modification

Ubiquitination is a dynamic and reversible post-translational modification that primarily mediates substrate degradation via the 26S proteasome, while also regulating signal transduction, cell cycle, and DNA damage repair (17, 18). The process involves three classes of enzymes.: E1 activates ubiquitin, E2 conjugates it, and E3 ubiquitin ligases confer substrate specificity by catalyzing ubiquitin attachment to target proteins (19). The human genome encodes approximately 600–1000 E3 ligases but only about 40 E2 enzymes, underscoring the central role of E3 ligases in substrate recognition (20). The fate of ubiquitinated proteins depends on the ubiquitin chain linkage type. K48-linked chains target proteins for proteasomal degradation, whereas K63-linked chains mediate signaling and DNA repair. Other linkage types (K6, K11, K27, K29, K33, M1) are involved in mitophagy, immune regulation, and cell cycle control (21–23).

Unlike ubiquitination, deubiquitination is mediated by DUBs that remove ubiquitin chains from substrate, regulating protein homeostasis and signal transduction (24). The DUB family comprises six major classes: ubiquitin-specific proteases (USPs), ubiquitin C-terminal hydrolases (UCHs), Machado–Joseph disease proteases (MJDs), ovarian tumor proteases (OTUs), motif interacting with ubiquitin-containing novel DUB family (MINDY), and JAB1/MPN/MOV34 metalloenzymes (JAMMs) (25). DUBs regulate cellular processes through three core mechanisms. First, they maintain protein stability: by removing K48-linked polyubiquitin chains; for example, USP9X enhances Wnt signaling by deubiquitinating and stabilizing β-catenin (26). Second, they edit signaling pathways by trimming ubiquitin chains; OTUD1 negatively regulates inflammatory responses by hydrolyzing K63-linked ubiquitin chains on signaling factors, including linear ubiquitin chain assembly complex (LUBAC), within the nuclear factor kappa-B (NF-κB) pathway (27), whereas CYLD suppresses NF-κB signaling and alleviates inflammation by deubiquitinating RIP1 (28). Third, they repair cellular damage by stabilizing key DNA repair proteins—such as USP11, which deubiquitinates and stabilizes BRCA1—or by regulating cellular stress response pathways. Through these actions, DUBs terminate ubiquitin signals or edit ubiquitin chain configurations. Acting in concert with E3 ligases, DUBs maintain the dynamic equilibrium of ubiquitination—where E3 ligases “label” proteins and DUBs “delabel” them—ensuring precise control of protein stability, activity, and cellular function (29).

## 3 Regulatory roles of E3 ubiquitin ligases and DUBs in T2DM

Within the intricate network of metabolic disturbances characteristic of T2DM, the stability of protein function and the precise regulation of signaling pathways are critically important. This delicate balance primarily relies on the dynamic and reversible process of ubiquitination modification, wherein E3 ubiquitin ligases are responsible for “tagging” specific substrate proteins for degradation, while their counterparts—DUBs—counterbalance this process by “removing” these tags. E3 ubiquitin ligases and DUBs play central roles in the pathogenesis of T2DM by precisely regulating the stability and function of proteins involved in key disease-related pathways. Consequently, dysregulation of specific E3 ubiquitin ligases or DUBs at critical metabolic nodes provides a direct mechanistic link between molecular perturbations and the progression of T2DM. **Table 1** systematically elucidates the pivotal roles of ubiquitination in T2DM from a bidirectional regulatory perspective. The table encompasses both protein degradation-driving mechanisms mediated by E3 ubiquitin ligases and protein-stabilizing protective mechanisms mediated by DUBs. It specifies the molecular targets of these two enzyme classes across key metabolic organs—including the liver, skeletal muscle, adipose tissue, and pancreas—their specific effects on substrate protein stability, activity, and subcellular localization, and their downstream pathophysiological consequences, such as IR, chronic inflammation, and β-cell dysfunction. Through juxtaposition and comparative analysis, this table presents a multilayered, cross-tissue view of the dynamic regulatory network governed by ubiquitination. It not only delineates the roles of individual enzyme-substrate axes in disease pathogenesis but also highlights the disruption of the delicate balance between E3 ligases and DUBs—where excessive driver ubiquitination coincides with relatively insufficient protective deubiquitination—thereby forming a molecular network basis for systemic metabolic dysregulation in T2DM. Importantly, this framework provides a clear target map for the development of precise therapeutic interventions.

**Table 1:** Molecular targets and pathological regulatory functions of key E3 Ligases and DUBs in T2DM.

Enzyme class	Enzyme name	Tissue specificity	Molecular target	Functional outcome	Therapeutic implication	References
E3 ubiquitin ligase	MG53	Muscle cell	IRS1	Promotes IR	Inhibiting its activity may improve skeletal muscle insulin sensitivity	(31)
	TRIM32	Hepatocyte	INSR	Promotes IR	Inhibiting its activity may improve hepatic insulin sensitivity	(34)
		Pancreatic β-cell	Akt-mTOR pathway	Promotes β-cell death	Inhibiting its activity may attenuate high glucose-induced β-cell death	(44)
		Podocytes	Akt-GSK-3β-Nrf2 Pathway	Alleviates oxidative damage in diabetic nephropathy	Enhancing its activity may alleviate podocyte oxidative damage	(48)
	MuRF1	Muscle cell	Akt	Influences glucose metabolism	Inhibiting its activity may improve skeletal muscle glucose metabolism	(36)
	TRIM21	Hepatocyte	PEPCK1, FASN	Ameliorates hepatic glucose and lipid dysregulation	Enhancing its activity may reduce hepatic gluconeogenesis and lipid accumulation	(39)
	FBXW7	Hepatocyte	Fetuin-A	Maintains hepatic glucose homeostasis and insulin sensitivity	Enhancing its activity may improve insulin sensitivity	(40)
	DDB2	Hepatocyte	KMT2A	Suppresses adipogenic programs and alleviates hepatic lipid accumulation	Enhancing its activity may alleviate hepatic steatosis	(42)
	March5	Pancreatic β-cell	Trim28	Promoting insulin gene transcription and glucose-stimulated insulin secretion	Enhancing its activity may restore β-cell function in diabetes	(43)
	SMURF1	β-cell	Stat3	Restricts β-cell proliferation	Inhibiting its activity may promote β-cell regeneration	(45)
		Bone marrow mesenchymal stem cell	PKG2	Inhibits osteogenic differentiation, linked to diabetes-associated osteoporosis	A potential therapeutic target for diabetes-associated osteoporosis	(50)
	Cbl	β-cell	EGFR-ERK signaling, dILP	Regulates insulin gene transcription	Enhancing its activity may promote insulin gene expression	(46)
	RNF186	Hepatocyte	JNK	Exacerbates ER stress and promotes inflammation and IR	Inhibiting its activity may reduce inflammation	(47)
	TRIM28	Muscle cell	?	Regulates mitochondrial function and autophagy (knockout shows no systemic metabolic abnormalities)	Unclear	(49)
	RNF167	Cardiac tissue	?	Disrupts protein homeostasis, exacerbates diabetic cardiomyopathy	Inhibiting its activity may ameliorate protein homeostasis in cardiomyocytes	(51)
	WWP2	Vascular endothelial cells	DDX3X	Alleviates ER stress and apoptosis, protects endothelial function	Enhancing its activity may protect vascular endothelial function	(52)
DUBs	USP25	Adipocytes	GLUT4	Maintains glucose uptake	Enhancing its activity may maintain glucose uptake	(53)
		Hepatocyte	PPARα	Promotes fatty acid oxidation	Enhancing its activity may promote fatty acid oxidation	(55)
		Podocyte	SMAD7	Protects against inflammation and apoptosis	Enhancing its activity may inhibit podocyte inflammation and apoptosis	(68)
	OTUD3	Adipocyte	PPARδ	Maintains energy metabolism	Enhancing its activity may maintain energy homeostasis	(54)
		Retinal pigment epithelium cell	PPARγ	Exerts anti-inflammatory and antioxidant effects	Enhancing its activity may exert anti-inflammatory and antioxidant effects (rs78466831 is a T2DM risk locus)	(70, 71)
	OTUD5	Hepatocyte	VDAC2	Maintains mitochondrial membrane potential and structural integrity	Enhancing its activity may protect mitochondrial function	(56)
		Podocyte	TAK1	Inhibits inflammation	Enhancing its activity may inhibit inflammatory pathways in podocytes	(69)
	OTUB1	Hepatocyte	TRAF6	Suppresses hepatic lipid metabolism	Restoring its expression may reduce hepatic steatosis	(57)
		Hepatocyte	YWHAB	Inhibits hepatic gluconeogenesis	Enhancing its activity may inhibit gluconeogenesis	(63)
	USP2	Hepatocyte	PPARγ	Promotes hepatic lipid accumulation	Inhibiting its activity may reduce hepatic steatosis	(58)
	USP15	Hepatocyte	FABP, perilipins	Promotes hepatic lipid accumulation and inflammatory responses	Inhibiting its activity may alleviate hepatic steatosis and inflammation	(59)
		Muscle cell	DRP1, AKT	Cooperates with USP30 to activate DRP1 and inhibit AKT signaling	Inhibiting its activity may improve mitochondrial function and insulin sensitivity	(61)
	USP30	Muscle cell	DRP1, AKT	Cooperates with USP15 to activate DRP1 and inhibit AKT signaling	Inhibiting its activity may improve mitochondrial function and insulin sensitivity	(61)
	USP7	Hepatocyte	FoxO1	Reduces hepatic gluconeogenesis	Enhancing its activity may reduce hepatic glucose output	(62)
		β cell	NGN3	Promotes differentiation of functional β-cells	Enhancing its activity may promote β-cell regeneration	(66)
	USP14	Hepatocyte	CBP	Enhances hepatic gluconeogenesis	IU1 (USP14 inhibitor) suppresses hepatic gluconeogenesis and reduces blood glucose	(64)
		Neuron	ERK1/2 pathway	Contributes to diabetic neuropathy	IU1 may improve cognitive dysfunction	(79)
	USP11	Prostate cancer cells	PTEN	Forms a PTEN-USP11-PI3K-FOXO positive feedback loop to maintain tumor suppression	Role in T2DM unclear	(65)
	USP5	Pancreatic β cells	Gαs	Modulating insulin secretion and β-cell proliferation	Enhancing its activity may preserve β-cell mass and function	(67)
	UCHL1	Sensory neurons	IRS1	Loss of UCHL1 induces neuronal insulin resistance and diabetic sensory neuropathy	CUL1 inhibitors may rescue UCHL1 loss-of-function	(72)
	USP10	Cardiomyocytes	Notch1	Suppresses myocardial fibrosis and apoptosis	Enhancing its activity may inhibit myocardial fibrosis and apoptosis	(73)
	USP18	Cardiomyocytes	Notch1	Activates AKT signaling, inhibits high glucose-induced oxidative stress, inflammation, and mitochondrial damage	Enhancing its activity may inhibit oxidative stress and inflammation	(74)
	OTUD1	Vascular endothelial cells	β-catenin	Impairs angiogenesis and delays wound healing	Inhibiting its activity may promote angiogenesis and wound healing	(75)
		Intestinal immune cells	RIPK1	Inhibits NF-κB and reduces inflammation	Potential anti-inflammatory target	(76)
		Hepatocyte	NRF2	Stabilizes antioxidant pathway; mitigates oxidative stress	Role in diabetic liver unclear	(77)

### 3.1 Regulatory role of E3 ubiquitin ligases in T2DM

**Figure 2:**
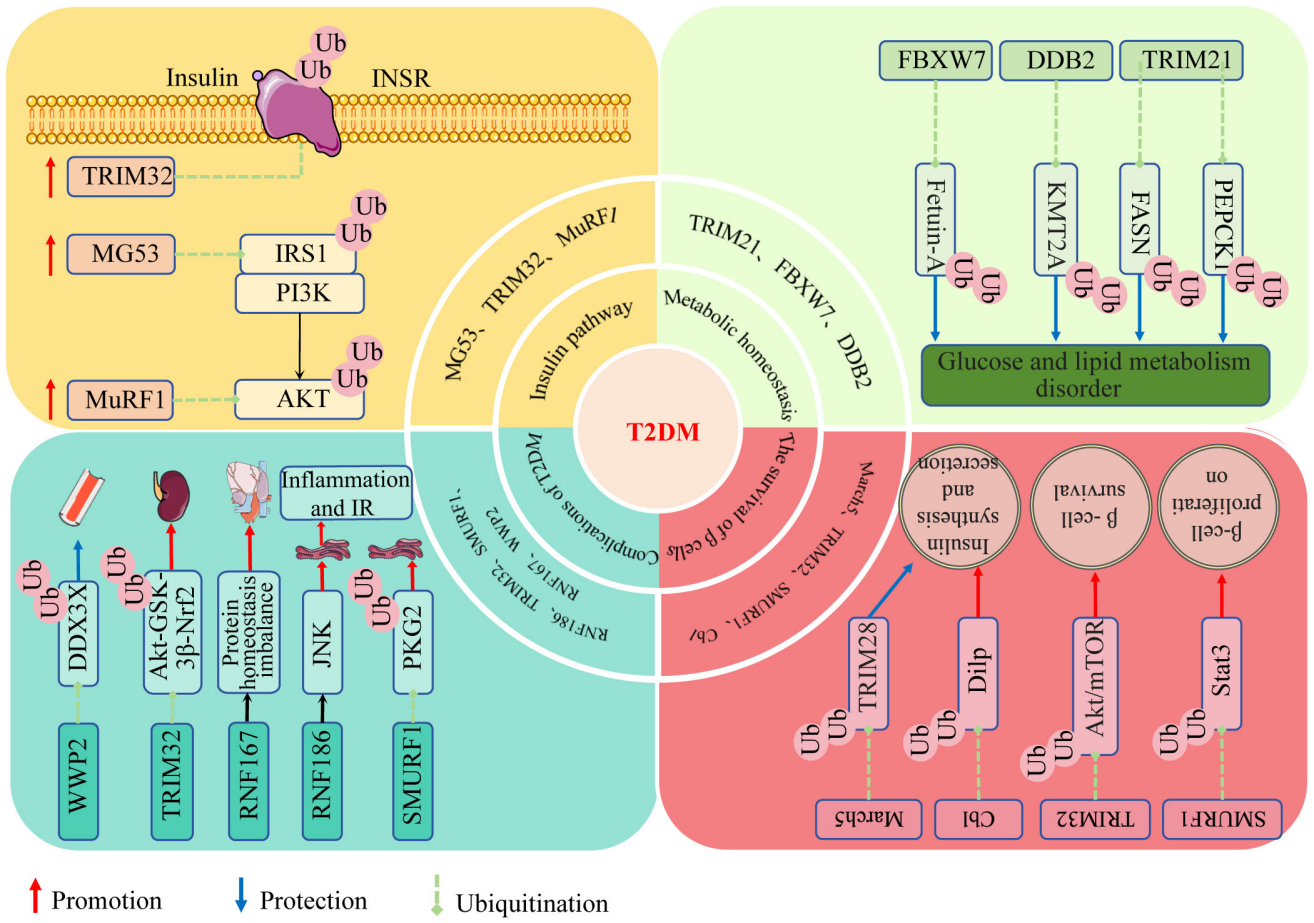
Modular regulatory network of E3 ubiquitin ligases in the pathological progression of T2DM. This figure illustrates the mechanisms by which different families of E3 ubiquitin ligases regulate the pathological progression of T2DM through four core dimensions. ① Yellow region: Regulation of the classical insulin signaling axis. TRIM32 targets INSR, MG53 targets IRS1, and MuRF1 targets AKT, collectively promoting insulin resistance. ② Pale green region: Regulation of metabolic homeostasis. FBXW7 targets Fetuin-A, DDB2 targets KMT2A, and TRIM21 targets FASN and PEPCK1, thereby alleviating glucose and lipid metabolic dysregulation. ③ Red region: Regulation of pancreatic β-cell proliferation and survival. SMURF1 targets Stat3 to restrict β-cell proliferation; TRIM32 targets the Akt/mTOR pathway to promote β-cell death; Cbl targets dILP to inhibit insulin biosynthesis; March5 targets TRIM28 to promote insulin gene transcription and glucose-stimulated insulin secretion. ④ Blue-green region: Mediation of diabetic complications. WWP2 targets DDX3X to alleviate ER stress and apoptosis, thereby protecting endothelial function; TRIM32 targets the Akt-GSK3β-Nrf2 axis to reduce oxidative damage in diabetic nephropathy; RNF167 disrupts protein homeostasis and exacerbates diabetic cardiomyopathy; RNF186 targets JNK to exacerbate ER stress promoting the release of inflammatory cytokines, which in turn induces IRS1 phosphorylation, ultimately leading to peripheral insulin resistance; SMURF1 targets PKG2 to inhibit osteogenic differentiation, which is associated with diabetes-related osteoporosis. Red arrows indicate promoting effects, blue arrows represent protective effects, and green dashed lines denote ubiquitination events.

As the “molecular determinant” of substrate specificity in the ubiquitination process, E3 ubiquitin ligases precisely regulate the ubiquitination modification of target proteins through selective substrate recognition. In doing so, they determine whether substrates undergo proteasomal degradation or experience functional modulation through non-degradative ubiquitination. This regulatory mechanism is central to key physiological processes such as insulin signaling, glucose and lipid metabolic homeostasis, and pancreatic β-cell dysfunction. To systematically elucidate the role of E3 ligases in T2DM pathogenesis, **Figure 2** presents an integrated systems biology perspective, illustrating how multiple E3 ligases collectively form coordinated, modular molecular networks that drive the pathological progression of T2DM. This systems-level perspective enables us to transcend the limitations of individual molecular functions and to comprehensively delineate the synergistic regulatory mechanisms of E3 ligases in the pathophysiological progression of T2DM from a network-regulation standpoint.

#### 3.1.1 Precise regulation of insulin signaling by E3 ligases

IR is a core pathophysiological mechanism underlying metabolic disorders across multiple tissues in the body (30). Its development is a multifactorial and multistage process in which post-translational protein modifications—particularly ubiquitin-mediated degradation driven by E3 ubiquitin ligases—play crucial regulatory roles. Through precise regulation of the stability of key components within the insulin signaling cascade, E3 ligases profoundly influence signal transduction efficiency and thereby play a pivotal role in the onset and progression of IR. In skeletal muscle, a major insulin-sensitive tissue, MG53 (also known as Tripartite motif-containing protein 72 [TRIM72]) exerts prominent regulatory functions. As an E3 ubiquitin ligase primarily expressed in skeletal muscle, the core mechanism of action of MG53 lies in its selective recognition and ubiquitin-dependent degradation of insulin receptor substrate 1 (IRS1). IRS1 serves as a critical adaptor protein within the insulin signaling pathway, and the stability of its protein levels is essential for effective downstream signal transduction. Targeted disruption of IRS1 by MG53 directly interrupts normal insulin signal transduction, thereby establishing MG53 as a pivotal molecular hub linking skeletal muscle-specific IR (31). Based on these mechanistic insights, researchers have developed innovative therapeutic strategies aimed at modulating this interaction. Small-molecule compounds or peptide-based therapeutics that specifically disrupt the physical interaction between MG53 and IRS1, may effectively prevent IRS1 ubiquitination and subsequent proteasomal degradation. Experimental studies have demonstrated that this intervention strategy significantly restores IRS1 protein expression in C2C12 mouse myoblast-derived myotube models, thereby improving cellular insulin sensitivity. Notably, a novel MG53-IRS1 interaction inhibitor, MID-00935, has demonstrated remarkable therapeutic potential in high-fat diet-induced obese mouse models and effectively alleviated systemic IR. These findings identify MID-00935 as a promising lead compound for future drug development (32, 33). Clinical and translational evidence assessment: current studies on MG53 are primarily based on C2C12 cell lines and high-fat diet-induced obese mouse models. Large-scale clinical cohort validation directly linking MG53 expression levels in human skeletal muscle to insulin sensitivity is currently lacking. The clinical translation of MID-00935 still requires phase I/II trials to confirm its safety and efficacy.

In addition to MG53, other E3 ubiquitin ligases are also integral components of the regulatory network governing IR across various metabolic tissues. For instance, TRIM32, another crucial E3 ubiquitin ligase, plays a pivotal role in the development of IR in skeletal muscle and liver tissues. Unlike MG53, which targets the upstream adaptor protein IRS1, TRIM32 acts at the receptor level by directly promoting the ubiquitination and degradation of the insulin receptor (INSR). The insulin receptor serves as the initial gateway for insulin signal transduction, and its abundance on the cell membrane surface of target tissues—such as the liver—directly determines insulin responsiveness. Therefore, excessive TRIM32 activity directly impairs hepatic insulin responsiveness and strongly promotes the development of diet-induced hepatic IR (34). Downstream of the insulin receptor, activation of the serine/threonine kinase AKT is critical for multiple metabolic processes, including promotion of glucose uptake and inhibition of gluconeogenesis (35). Certain E3 ubiquitin ligases have also been shown to regulate insulin signaling by targeting AKT, thereby influencing its stability and functional activity. For example, the muscle-specific RING finger protein 1 (MuRF1), an E3 ligase, regulates AKT stability by recognizing and ubiquitinating specific phosphorylation sites on AKT, such as Ser-473. This regulatory activity directly affects AKT-mediated glucose metabolism and anabolic processes in skeletal muscle, thereby modulating IR at a more refined molecular level (36). Collectively, these findings indicate that E3 ubiquitin ligases regulate key components of the insulin signaling pathway through multi-level mechanisms. This not only deepens our understanding of the molecular basis of IR but also identifies potential novel targets for the development of precision therapeutic strategies.

#### 3.1.2 Central role of E3 ubiquitin ligases in metabolic homeostasis

Within the intricate regulatory network governing metabolic diseases, the role of E3 ubiquitin ligases extends far beyond the modulation of insulin signaling pathways. Their regulatory scope encompasses multiple essential physiological processes, including lipid metabolism and glucose homeostasis, thereby establishing them as central determinants of metabolic balance. Research indicates that TRIM21, a member of the class IV TRIM family, plays a pivotal role in mitigating systemic metabolic disturbances. In obese diabetic mouse models, TRIM21 overexpression significantly alleviates multiple metabolic abnormalities, including glucose intolerance, IR, hepatic steatosis, and dyslipidemia. Phosphoenolpyruvate carboxykinase 1 (PEPCK1) is the rate-limiting enzyme in gluconeogenesis, whereas fatty acid synthase (FASN) is the key enzyme in fatty acid synthesis (37, 38). Further mechanistic analyses revealed that TRIM21 specifically recognizes and promotes the ubiquitin-dependent degradation of the rate-limiting metabolic enzymes PEPCK1 and FASN through its E3 ligase activity. By simultaneously downregulating the protein levels of both enzymes, TRIM21 effectively ameliorates hepatic glucose and lipid metabolic dysregulation in T2DM models, thereby establishing itself as an important regulator of liver metabolism in T2DM (39). In addition to TRIM21, other E3 ligases contribute uniquely to hepatic metabolic homeostasis through different molecular targets. As a member of the F-box protein family, FBXW7 directly binds to the hepatokine fetuin-A, inducing its ubiquitination and subsequent proteasomal degradation. Fetuin-A is a known liver-secreted glycoprotein whose circulating levels are elevated in IR and metabolic syndrome. FBXW7-mediated degradation of fetuin-A is an important mechanism for maintaining hepatic glucose homeostasis and insulin sensitivity (40). In addition to its role in fetuin-A degradation, FBXW7 also participates in lipid metabolism through its cytoplasmic isoform FBXW7β. FBXW7β functions as an E3 ubiquitin ligase for fatty acid synthase (FASN), promoting its ubiquitination and degradation by recognizing a conserved phospho-degron motif within FASN (41). This regulatory axis is closely associated with T2DM, as excessive *de novo* lipogenesis can lead to lipotoxicity and IR. Notably, CSN6 stabilizes FASN by promoting FBXW7β autoubiquitination and degradation, thereby enhancing lipogenesis; conversely, loss-of-function mutations of FBXW7β also impair FASN degradation, resulting in abnormal lipid accumulation. Although these findings originate from cancer research, dysregulation of the FBXW7β-FASN axis likely contributes to the aberrant lipid accumulation observed in diabetic adipose tissue and liver, linking ubiquitination-mediated FASN regulation to T2DM-associated lipotoxicity and inflammatory complications. Future studies could explore whether targeting this axis could ameliorate lipotoxicity and improve metabolic outcomes in T2DM.

Similarly, DNA damage-binding protein 2 (DDB2), another E3 ubiquitin ligase, exerts metabolic regulatory functions by targeting the histone methyltransferase KMT2A for ubiquitination and proteasomal degradation. KMT2A is a key epigenetic regulator of adipogenesis-related gene expression. By promoting KMT2A degradation, DDB2 suppresses downstream adipogenic programs, thereby alleviating hepatic lipogenesis, reducing lipid accumulation, and inhibiting lipid metabolic disturbances in T2DM. Consequently, DDB2 has been identified as a critical regulator of lipid metabolic balance (42). Notably, E3 ubiquitin ligase-mediated metabolic regulation is not limited to the liver but instead operates through a coordinated, multi-organ regulatory network. In the skeletal muscle, another crucial insulin-sensitive tissue, the muscle-specific E3 ligase MuRF1 (TRIM63) has been demonstrated to influence glucose metabolism through mechanisms such as modulation of AKT stability (36), thereby complementing hepatic E3 ligases within systemic metabolic regulation. In addition, specific E3 ligases participate in localized metabolic regulation within other metabolically active tissues, including the adipose tissue and the pancreas. Collectively, these findings underscore the extensive and complex regulatory roles of E3 ubiquitin ligases in metabolic disease. Tissue-specific mechanisms converge across metabolic organs to establish an integrated ubiquitin-dependent network that governs systemic homeostasis.

#### 3.1.3 Molecular link between E3 ubiquitin ligase and β-Cell dysfunction

In the regulatory network governing pancreatic β-cell function, E3 ubiquitin ligases precisely modulate insulin synthesis, secretion, and cellular homeostasis through multidimensional mechanisms, thereby serving as pivotal regulatory hubs for maintaining both β-cell function and quantity. March5, an E3 ligase localized to the mitochondrial outer membrane, has been identified as a core regulatory factor essential for preserving β-cell function. Its mechanism involves the selective recognition and ubiquitin-mediated degradation of TRIM28, thereby relieving TRIM28-mediated transcriptional repression of the Kindlin-2 protein. Restoration of Kindlin-2 expression directly stabilizes MafA, a key transcriptional regulator of insulin gene expression, ultimately forming the March5-TRIM28-Kindlin-2-MafA signaling axis that promotes insulin gene transcription and glucose-stimulated insulin secretion (43). TRIM32 exhibits dual regulatory properties in β cells. Under high-glucose stress conditions, TRIM32 abnormally promotes excessive activation of autophagic flux through the Akt/mTOR signaling pathway, ultimately leading to autophagy-associated β-cell death. This process not only directly impairs β-cell mass but also indirectly weakens insulin secretory capacity by compromising cellular viability, thereby contributing to the pathogenesis of T2DM (44). SMAD-specific E3 ubiquitin protein ligase 1 (Smurf1) plays a negative regulatory role in maintaining β-cell homeostasis. Its core function is the selective recognition and ubiquitin-mediated degradation of signal transducer and activator of transcription 3 (Stat3), thereby directly blocking the JAK-Stat3 signaling pathway—a critical pathway for β-cell proliferation—and consequently restricting the self-renewal capacity of β-cells (45). Notably, this inhibitory process is regulated by the dynamic equilibrium of the cytoskeletal adaptor protein Talin-1: Talin-1 directly binds to Smurf1 and suppresses its E3 ligase activity, thereby stabilizing Stat3 protein levels and ultimately promoting β-cell proliferation. This dynamic regulatory network, formed by the Talin-1–Smurf1–Stat3 axis, provides a crucial molecular mechanism for maintaining β-cell population homeostasis. The Casitas B-lineage lymphoma (CBL) family of E3 ligases exerts evolutionarily conserved regulatory functions in modulating insulin synthesis. Studies using a Drosophila model revealed that Cbl directly influenced the expression levels of insulin-like peptides by modulating the epidermal growth factor receptor (EGFR)-extracellular signal-regulated kinase (ERK) signaling pathway (46). This regulatory mechanism was further validated in mammalian systems. Experiments in rat pancreatic β cells confirmed that Cbl also participates in the transcriptional regulation of insulin genes through the EGFR-ERK signaling pathway, demonstrating the conservation of this regulatory pathway across species (46). Collectively, E3 ligases regulate β-cell transcriptional activity (March5/MafA), cellular autophagy (TRIM32), proliferation capacity (Smurf1/Stat3), and intracellular signaling (Cbl/EGFR-ERK) by selectively targeting critical molecular substrates. Dysregulation of this tightly coordinated network may lead to insulin secretion defects and β-cell mass reduction, thereby playing a central role in the pathogenesis of diabetes.

#### 3.1.4 Role of E3 ubiquitin ligases in the T2DM complication network

In the pathogenesis of T2DM, metabolic inflammation and tissue-specific pathological changes are intricately intertwined, forming a complex pathological network. Within this context, E3 ubiquitin ligases function as central molecular hubs by precisely regulating key inflammatory and stress-signaling pathways. Different E3 ligases often exert opposing effects on metabolic inflammation. Pro-inflammatory E3 ligases, such as RNF186, exacerbate endoplasmic reticulum stress by activating the c-Jun N-terminal kinase (JNK) signaling pathway, thereby promoting the production and release of inflammatory cytokines, including tumor necrosis factor-α (TNF-α) and interleukin-6. These inflammatory factors further induce IRS1 phosphorylation, ultimately leading to IR in the peripheral tissues and directly driving the development of T2DM (47). In contrast, certain E3 ligases exert anti-inflammatory effects. For example, TRIM32 enhances cellular antioxidant capacity by modulating the Akt/glycogen synthase kinase-3β (GSK-3β)/nuclear factor erythroid 2-related factor 2 (Nrf2) signaling axis under high-glucose conditions, thereby alleviating inflammatory responses and oxidative damage in podocytes during diabetic nephropathy and conferring cytoprotective effects (48). This “bidirectional regulation” underscores the precise control exerted by E3 ligases over the metabolic-inflammatory balance. Notably, the regulatory roles of E3 ligases are highly tissue-specific and mechanistically complex. Although TRIM28 participates in the regulation of mitochondrial function and autophagy in skeletal muscle, muscle-specific knockout of TRIM28 does not result in significant systemic metabolic abnormalities. This observation suggests that its function may be restricted by tissue context or compensated for by other E3 ligases or parallel signaling pathways (49). In the skeletal system, SMURF1 inhibits the osteogenic differentiation of mesenchymal stem cells through a dual pathway by ubiquitinating and promoting the degradation of cGMP-dependent protein kinase 2 (PKG2), while simultaneously activating endoplasmic reticulum stress. This mechanism may represent a key molecular basis underlying diabetes-associated osteoporosis (50). In cardiac tissues, upregulated expression of the E3 ligase RNF167, together with decreased proteasome activity under diabetic conditions, disrupts protein homeostasis in cardiomyocytes. This disruption may promote the accumulation of misfolded proteins, thereby exacerbating the pathological progression of diabetic cardiomyopathy (51). E3 ligases also contribute to the development of diabetic vascular complications. In vascular endothelial cells, WWP2 mediates K63-linked ubiquitination of the RNA helicase DDX3X and promotes its degradation. This process effectively alleviates high-glucose- and lipotoxicity-induced endoplasmic reticulum stress and apoptosis, thereby protecting endothelial function and highlighting the therapeutic potential of WWP2 as a protective factor against diabetic vasculopathy (52). Clinical and translational evidence assessment: The role of RNF167 in diabetic cardiomyopathy is primarily derived from animal models. Expression profiling and functional validation of RNF167 in human diabetic myocardial tissue have not yet been reported. In summary, E3 ubiquitin ligases regulate metabolic inflammation and multi-tissue pathological processes in T2DM and its complications through highly tissue-specific expression patterns and substrate recognition mechanisms.

### 3.2 DUBs: the molecular bridge Linking IR and β-cell dysfunction

**Figure 3:**
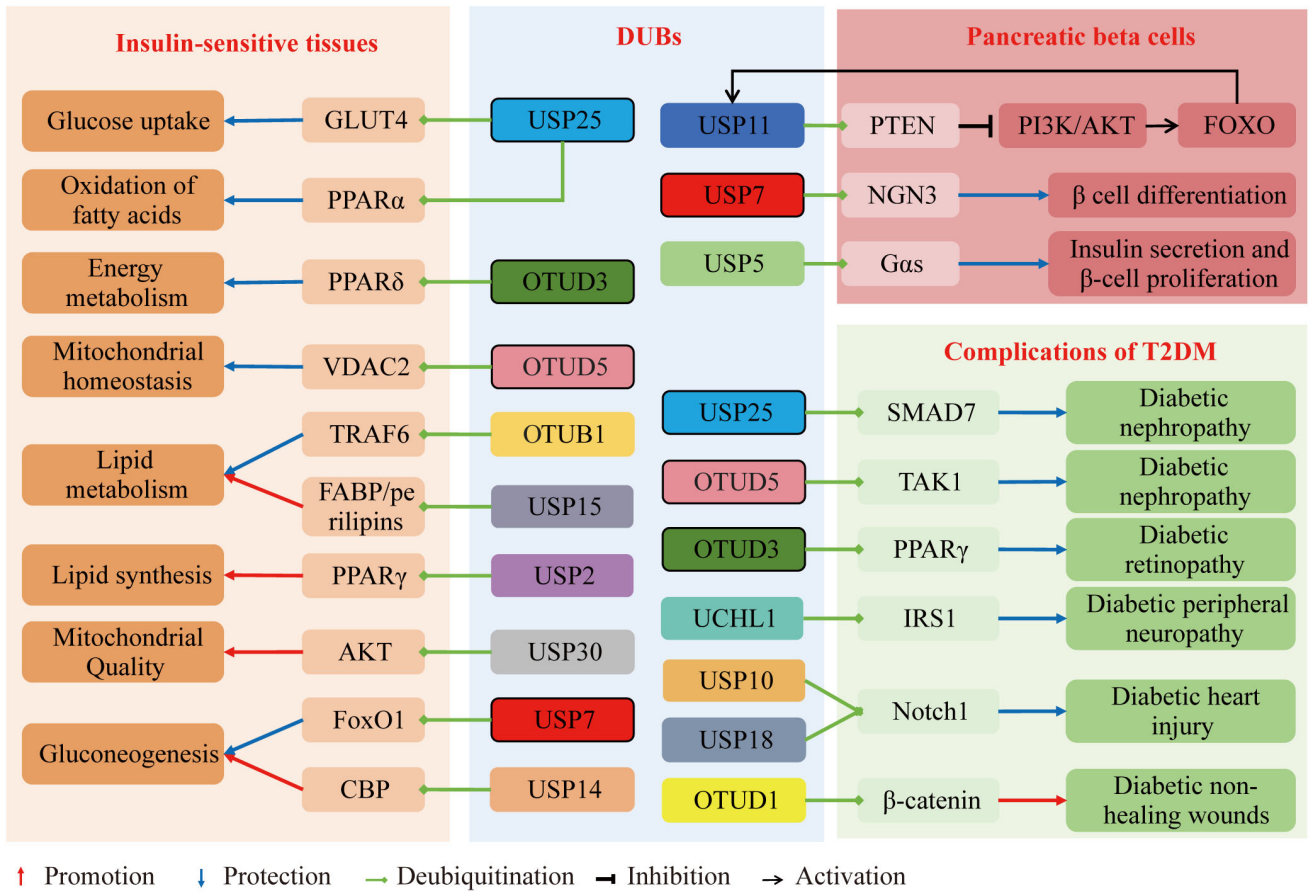
Multidimensional regulatory networks and multifunctional roles of DUBs in the pathological landscape of T2DM. The red arrows and blue arrows represent promotive and protective effects, respectively; solid green lines represent deubiquitination; black arrows denote activation; and black T-shaped arrows indicate inhibition.

DUBs, as key executors of reversible ubiquitination modifications, have emerged as central molecular hubs connecting peripheral IR with pancreatic β-cell dysfunction through their precise regulation of substrate protein stability and biological activity. Elucidating the bidirectional regulatory mechanisms underlying the two core pathological components of T2DM is crucial for a systematic understanding of disease-associated metabolic disorders. **Figure 3** systematically integrates the regulatory pathways of DUBs across multiple pathological contexts from three dimensions—”insulin-sensitive tissues,” “pancreatic β-cells,” and “diabetic complications”—while highlighting the functional diversity of individual DUBs across different physiological processes.

#### 3.2.1 DUBs in insulin-sensitive tissues: defending against IR

DUBs effectively maintain glucose homeostasis by directly stabilizing the key components of the insulin signaling pathway, thereby preserving the efficiency and fidelity of signal transduction. Within this regulatory network, USP25 positively regulates insulin-stimulated glucose uptake in adipose tissue by directly deubiquitinating and stabilizing the glucose transporter GLUT4, thereby preventing its proteasomal degradation (53). Meanwhile, OTUD3 operates at the transcriptional level by deubiquitinating and stabilizing metabolism-related transcription factors, such as peroxisome proliferator-activated receptor δ (PPARδ), thereby maintaining cellular energy metabolism activity. Importantly, mice with OTUD3 deficiency exhibit severe metabolic disturbances, including diet-induced obesity and IR, confirming its pivotal role in systemic energy homeostasis (54).

In addition to directly regulating the insulin signaling pathway, DUBs indirectly influence insulin sensitivity by modulating lipid metabolism. Lipid deposition and oxidative stress in the liver and adipose tissues are significant contributors to IR, and DUBs play crucial roles in these processes. Beyond its established function in glucose uptake, USP25 also exerts an important role in hepatic lipid metabolism. It interacts with PPARα, removing its K48-linked polyubiquitin chains to enhance PPARα stability, thereby promoting fatty acid oxidation and alleviating high-fat diet-induced hepatic lipid accumulation (55). Similarly, OTUD5 maintains mitochondrial membrane potential and structural integrity by stabilizing the mitochondrial outer membrane protein voltage-dependent anion channel (VDAC2), and its downregulation is closely associated with the progression of metabolic dysfunction-associated steatohepatitis (56). DUBs play opposing roles within the intricate regulatory network of lipid metabolism. OTUB1 serves as a critical suppressor of hepatic lipid metabolism by binding to and inhibiting TRAF6’s K63-linked ubiquitination, thereby blocking the activation of the ASK1-p38/JNK signaling pathway (57). In contrast, USP2 promotes the expression of lipid synthesis-related genes by deubiquitinating and stabilizing PPARγ, consequently exacerbating hepatic lipid accumulation (58). Further studies have revealed that under metabolic stress conditions, the expression levels of USP15 and USP30 are negatively correlated with peripheral insulin sensitivity. USP15 primarily enhances the stability of lipid metabolism-related proteins, such as FABP and perilipins, through deubiquitination, thereby promoting hepatic lipid accumulation and inflammatory responses (59). In contrast, USP30 disrupts mitochondrial quality control, leading to the accumulation of dysfunctional mitochondria and directly impairing normal insulin signal transduction (60). In-depth mechanistic investigations have demonstrated that overexpression of USP15 and USP30 coordinately regulates key signaling nodes by activating the mitochondrial fission protein DRP1, while simultaneously inhibiting the phosphorylation of AKT and its downstream substrate AS160 within the insulin-signaling pathway (61). This discovery provides important mechanistic insights into how DUBs impair insulin sensitivity under lipotoxic conditions.

In the regulation of glucose metabolism, DUBs maintain hepatic glucose output homeostasis through precise modulation of key gluconeogenic transcription factors. Notably, USP7 acts as a negative regulator of FoxO1 by mediating its deubiquitination while unexpectedly suppressing its transcriptional activity, thereby downregulating the expression of key gluconeogenic genes and ultimately reducing hepatic glucose output (62). Notably, OTUB1 is downregulated in type 2 diabetic livers, where it directly binds to and catalyzes the deubiquitination of YWHAB (14-3-3β), enhancing its stability and consequently inhibiting hepatic gluconeogenesis (63). Conversely, USP14 is upregulated under sustained endoplasmic reticulum stress and enhances hepatic gluconeogenesis by stabilizing CREB-binding protein (CBP), which antagonizes insulin action in the liver and disrupts systemic glucose homeostasis (64).

In summary, deubiquitinating enzymes—such as USP25, OTUD3, OTUD5, and OTUB1—maintain metabolic homeostasis in insulin-sensitive tissues through a highly coordinated regulatory network operating at multiple levels, including transcription factor stability, signal transduction, organelle function, and metabolic enzyme activity. Notably, many DUBs exhibit “multifunctional enzyme” characteristics, acting on distinct substrates within the same or different tissues to generate synergistic effects that collectively counteract IR.

#### 3.2.2 Role of DUBs in pancreatic β-cells: protective functions and survival

The functional integrity and survival of pancreatic β-cells play a decisive role in T2DM pathogenesis. Recent studies have revealed that DUBs regulate β-cell development, function, and survival through multiple molecular mechanisms, forming a sophisticated protective network. USP11 has been identified as a key deubiquitinase for the PTEN protein, which is involved in the β-cell signaling pathways. Mechanistic studies have demonstrated that USP11 directly binds to and deubiquitinates PTEN, effectively counteracting ubiquitination-mediated degradation and maintaining PTEN protein stability. This regulatory process subsequently inhibits the PI3K/AKT signaling pathway while promoting nuclear translocation and activation of FOXO transcription factors. Notably, activated FOXO factors enhance USP11 transcription, forming a self-reinforcing positive feedback loop (65). Although this regulatory mechanism has been well characterized in cancer biology, its relevance to pancreatic β-cell function and T2DM pathogenesis remains to be elucidated. Whether this USP11-PTEN-FOXO axis operates in β-cells and how it might influence insulin secretion or β-cell survival under IR are important questions for future investigation.

However, the specific pathophysiological significance of this finely tuned regulatory circuit under IR remains unclear. From a β-cell development and differentiation perspective, USP7 plays a pivotal role during pancreatic development. Mechanistic studies reveal that USP7 directly interacts with the transcription factor NGN3 to remove its ubiquitination modifications, thereby enhancing NGN3 stability and promoting directed differentiation of pancreatic endocrine lineages and the generation of functional β-cells (66). This discovery highlights the central role of USP7 in β-cell development. At the level of β-cell function regulation, USP5 has been identified as a crucial positive regulator of the Gαs/cAMP signaling pathway. Mechanistic studies indicate that USP5 collaborates with Tipe1 to specifically remove K48-linked polyubiquitin chains from the G protein subunit Gαs, preventing its proteasomal degradation and enhancing protein stability. This regulatory mechanism enhances cAMP pathway activation, thereby precisely modulating insulin secretion and β-cell proliferation processes, ultimately maintaining β-cell homeostasis and suppressing T2DM progression (67). In summary, DUBs such as USP11, USP7, and USP5 constitute a sophisticated regulatory network across multiple functional domains of β-cells through distinct molecular mechanisms.

#### 3.2.3 Role of DUBs in T2DM complications

Diabetes-related complications are the primary contributors to poor prognosis. DUBs intricately regulate key pathological processes such as inflammation, oxidative stress, and fibrosis, and are critically involved in the development of various diabetic complications. Multiple DUBs play protective roles in diabetic nephropathy. USP25, a major component of adipose-derived stem cell exosomes, directly interacts with SMAD7 and catalyzes its deubiquitination. This process prevents SMAD7 degradation via the ubiquitin-proteasome pathway, thereby enhancing SMAD7 stability and effectively suppressing high glucose-induced podocyte inflammation and apoptosis (68). Meanwhile, OTUD5 specifically removes K63-linked ubiquitin chains from TAK1 through its active site C224, inhibiting TAK1 phosphorylation and the downstream MAPK/NF-κB signaling pathways. This process negatively regulates high glucose/palmitate-induced inflammatory factor production in podocytes, protects podocyte function, and delays diabetic nephropathy progression (69).

OTUD3 is a key regulator of retinal homeostasis in diabetic retinopathy. Specifically, OTUD3 exerts anti-inflammatory and antioxidant effects by deubiquitinating PPARγ. However, under pathological conditions, pro-inflammatory TNF-α inhibits this protective pathway, impairing the OTUD3-PPARγ axis and exacerbating retinal damage (70). Genetic evidence further supports this mechanism, as OTUD3 (rs78466831) represents a risk locus for T2DM and may contribute to diabetic retinopathy development (71). Clinical and translational evidence assessment: OTUD3 (rs78466831) is a reported T2DM risk locus, providing direct human genetic evidence. However, its causal role in diabetic retinopathy still requires functional genomic studies for confirmation.

In the nervous system, UCHL1 regulates insulin signaling by deubiquitinating insulin receptor substrate 1 (IRS1), while antagonizing its ubiquitination and degradation of IRS1 by the E3 ligase Cullin1 (CUL1), thereby maintaining neuronal function in diabetic peripheral neuropathy (72). Previous studies have also identified multiple DUB-mediated protective pathways in the cardiovascular system. In diabetic myocardial infarction, the FSTL1-USP10-Notch1 axis constitutes a novel cardioprotective signaling pathway. USP10 serves as a key deubiquitinase, suppressing fibrosis and apoptosis by stabilizing Notch1 signaling (73). Similarly, USP18 protects against diabetes-induced cardiac injury by stabilizing Notch1 and promoting AKT signaling, thereby inhibiting high glucose-induced oxidative stress, inflammatory response, and mitochondrial damage in cardiomyocytes (74).

OTUD1 exhibits complex and context-dependent regulatory functions in diabetic complications. Notably, in the typical complication of diabetic wound healing, OTUD1 plays a negatively regulates angiogenesis by removing K63-linked polyubiquitin chains at the K496, K508, and K625 sites of β-catenin protein, thereby impairing its pro-angiogenic capacity of β-catenin and ultimately leading to endothelial dysfunction and delayed wound repair (75). This finding is particularly unique, as OTUD1 typically exerts protective effects in other non-diabetes-related pathological conditions. For instance, in inflammatory bowel disease models, OTUD1 inhibits NF-κB activation by deubiquitinating RIPK1, thereby reducing the production of pro-inflammatory cytokine production (76). Conversely, during hepatic ischemia-reperfusion injury, OTUD1 stabilizes to activate antioxidant pathways, mitigating oxidative stress and inflammation (77). These contrasting functional outcomes across different disease models highlight the complexity of OTUD1’s biological effects. However, the specific mechanisms by which OTUD1 regulates inflammatory networks in T2DM and its complications, and the critical points at which its function shifts from protective to detrimental, remain poorly understood further elucidation.

## 4 Targeting the ubiquitination system: a new frontier for precision therapy in T2DM

**Figure 4:**
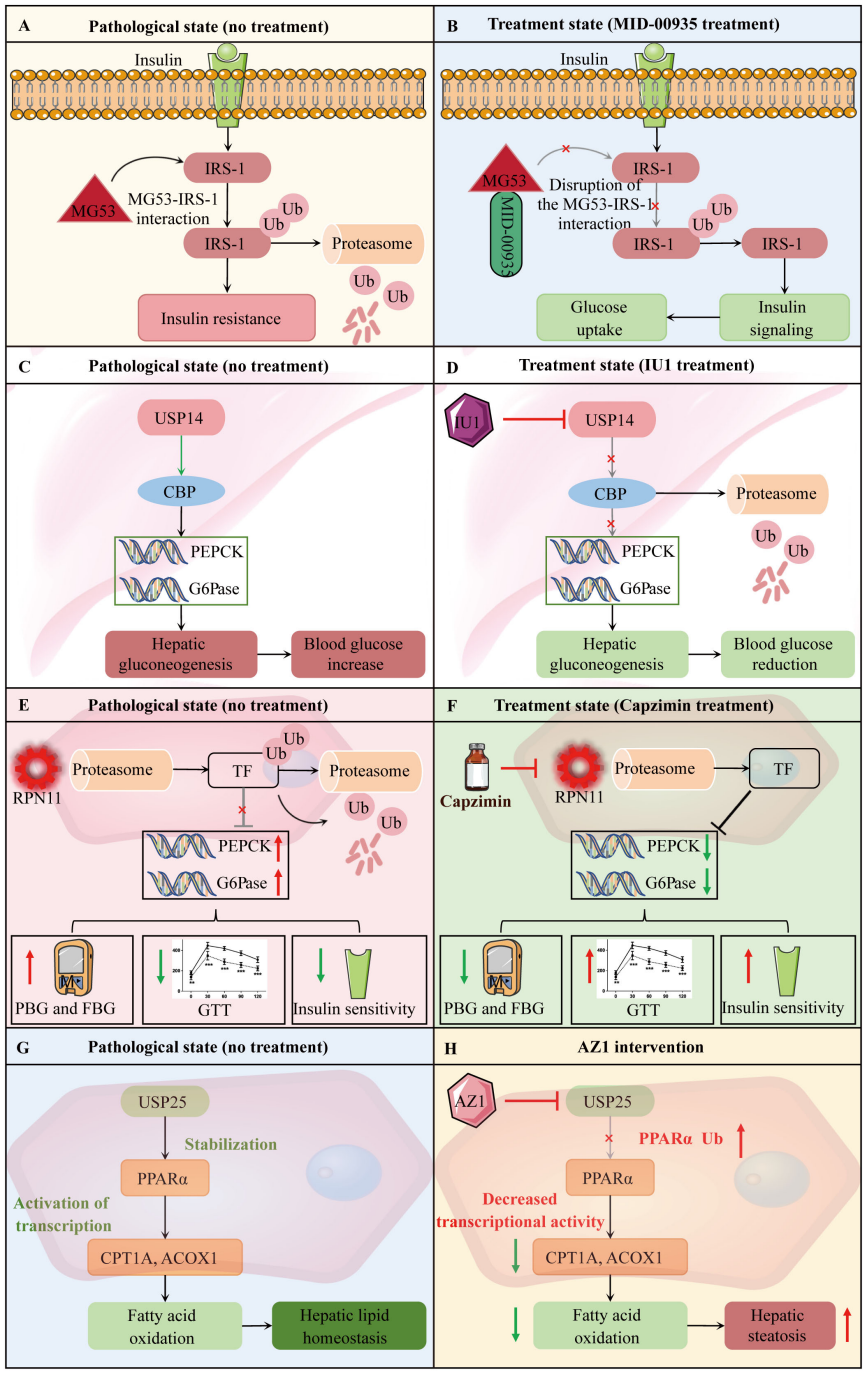
Network diagram of the mechanism of action of ubiquitination inhibitors in T2DM: integration of pathological state and pharmacological intervention. **(A)** Inhibitory mechanism of MG53-mediated insulin signaling: Under pathological conditions, MG53 abnormally binds IRS1 and promotes its ubiquitination and degradation, leading to insulin resistance. **(B)** Molecular mechanism of MID-00935 in improving insulin signaling: MID-00935 specifically disrupts the MG53-IRS1 interaction, restoring IRS1 stability, and insulin signal transduction. **(C)** Molecular mechanism of USP14 in promoting hepatic gluconeogenesis: Under pathological conditions, the deubiquitinating effect of USP14 maintains CBP protein stability, upregulates gluconeogenic gene expression, and induces hyperglycemia. **(D)** Molecular mechanism of IU1 in improving hepatic glucose metabolism: IU1 inhibits USP14, reduces CBP stability and gluconeogenic gene expression, thereby suppressing hepatic gluconeogenesis and lowering blood glucose levels. **(E)** Pathological Mechanism of RPN11 in diabetes: under such conditions, the enhanced deubiquitinating activity of RPN11 stabilizes substrate proteins, promoting diabetes-related phenotypes. **(F)** Molecular Mechanism of the RPN11 Inhibitor Capzimin in improving diabetic phenotypes: Capzimin improves glycemic control and insulin sensitivity by inhibiting RPN11 and downregulating substrate protein stability. **(G)** Pathological mechanism of USP25 in maintaining hepatic lipid homeostasis: under pathological conditions, USP25 overexpression maintains hepatic fatty acid oxidation by stabilizing PPARα, thereby ameliorating hepatic steatosis. **(H)** Mechanism by which the USP25 inhibitor AZ1 exacerbates hepatic steatosis: AZ1-mediated USP25 inhibition suppresses fatty acid oxidation and attenuates pathways including PPARα, leading to triglyceride accumulation and aggravated steatosis.

The ubiquitination system critically regulates insulin signaling, β-cell function, and systemic glucose-lipid homeostasis by controlling the stability and activity of key proteins. Consequently, developing small-molecule modulators that specifically target E3 ubiquitin ligases or DUBs has emerged as a promising strategy for precision intervention in T2DM. By designing compounds that selectively enhance or inhibit specific E3 ligases or DUBs, researchers aim to restore insulin sensitivity at the molecular level, protect β-cell function, and alleviate metabolic inflammation, thereby enabling disease-modifying interventions. In this context, various inhibitors targeting hyperactive components of the ubiquitination system have entered preclinical research pipelines. **Figure 4**, **Table 2** illustrate the mechanisms of four inhibitors (MID-00935, IU1, Capzimin, and AZ1) across two dimensions: “pathological/physiological state” and “pharmacological intervention.” This representation delineates the molecular pathways regulating ubiquitination and highlights the improvement of diabetic phenotypes under different metabolic conditions.

**Table 2:** The potential applications and mechanisms of molecular inhibitors targeting the ubiquitination system in T2DM.

Inhibitor	Chemical structure	Target	Potential functions and mechanisms	References
MID-00935	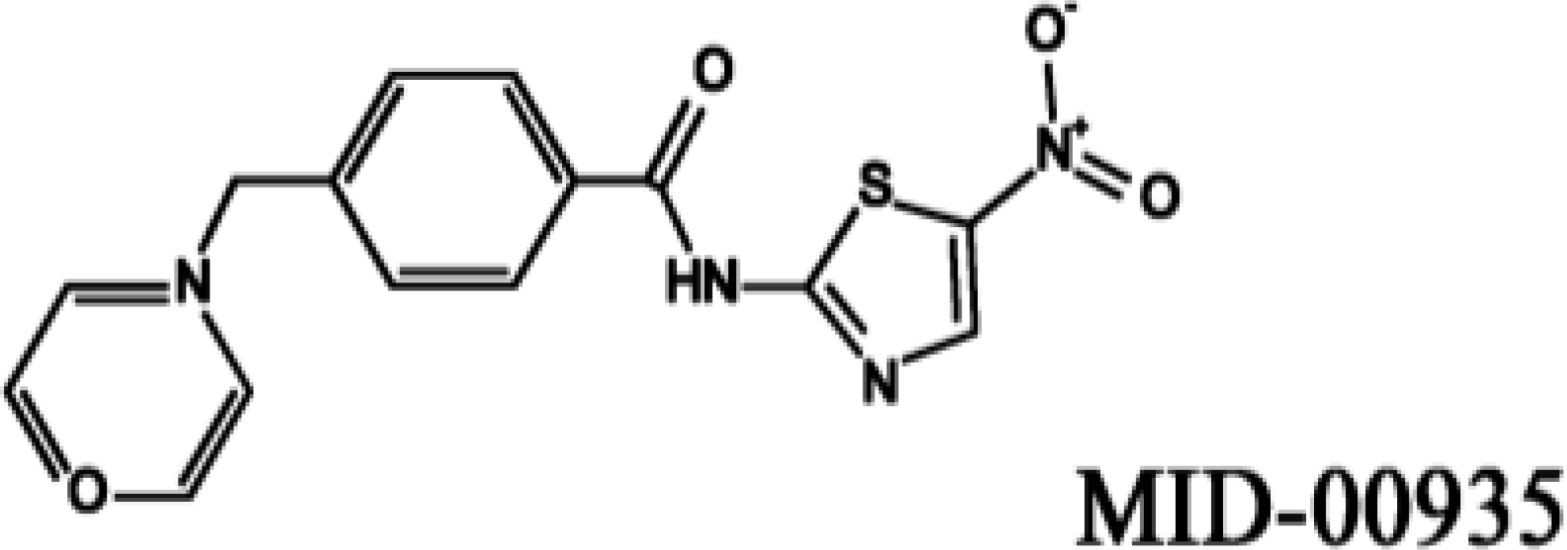	MG53	Disrupts MG53–IRS1 interaction, abrogates MG53-induced IRS1 ubiquitination and degradation, and improves insulin signaling in C2C12 myotubes	(33)
IU1	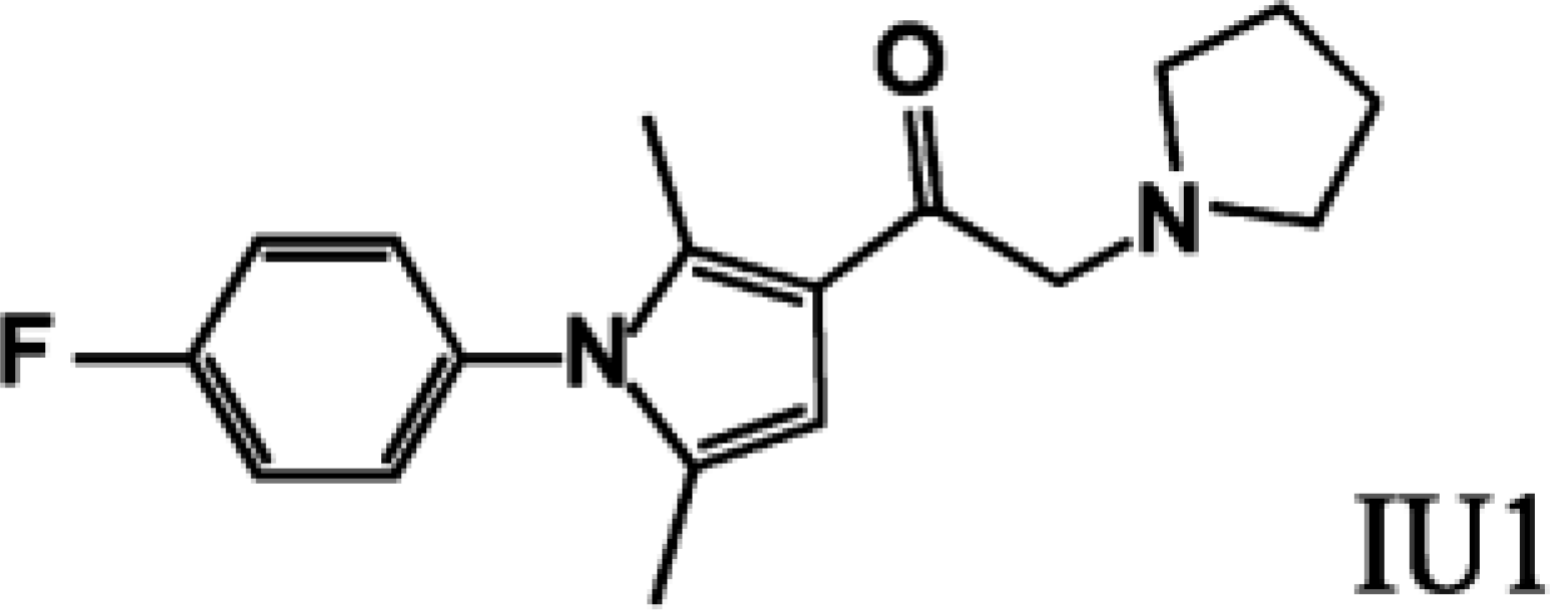	USP14	Suppresses hepatic gluconeogenesis and lowers blood glucose by blocking USP14/CBP/glucagon signaling	(67)
			Improves glucose homeostasis in T2DM mice by inhibiting the USP14/ERK1/2 pathway	(79)
Capzimin	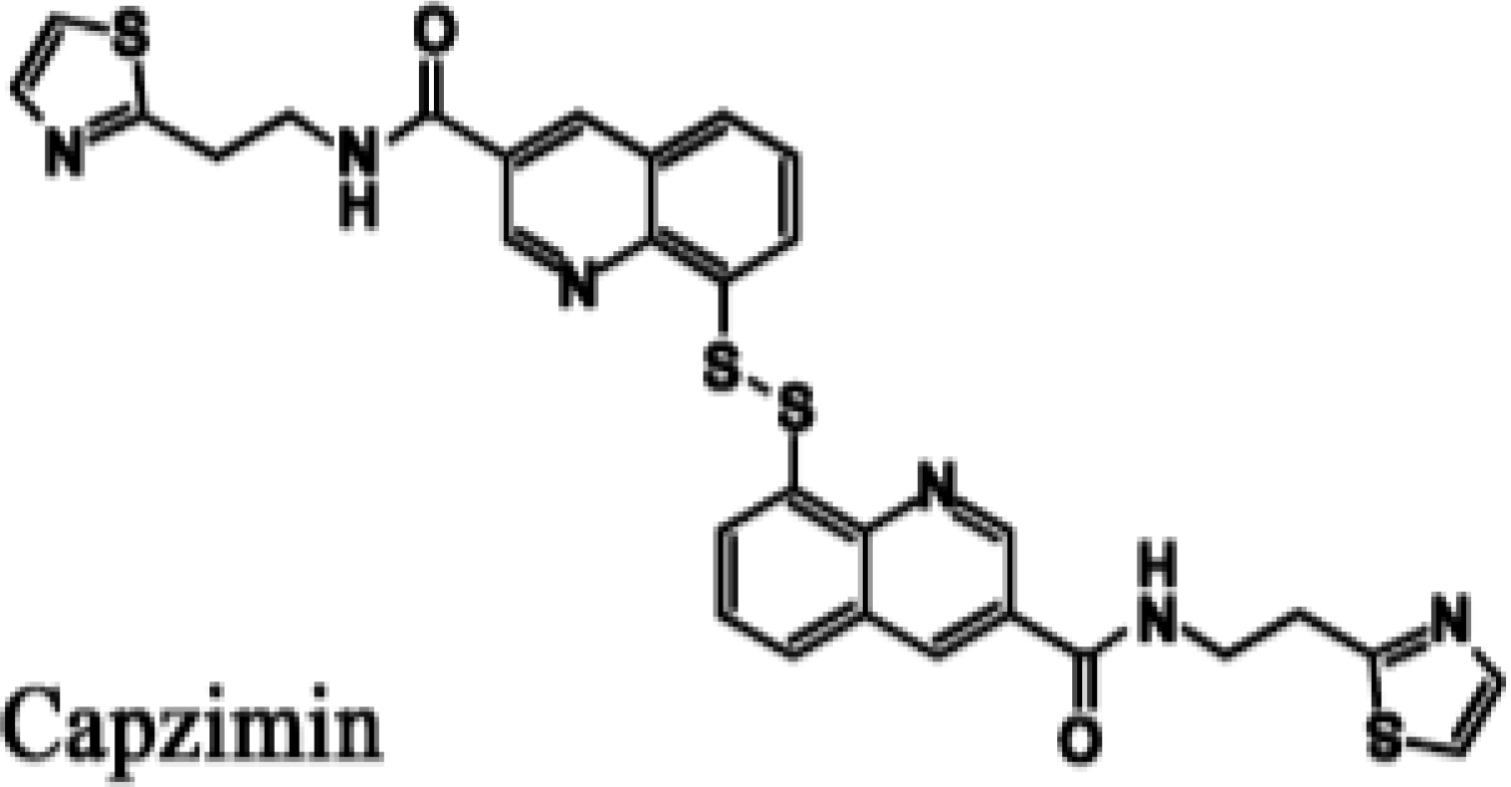	RPN11	Intraperitoneal injection significantly alleviates T2DM phenotypes, reduces blood glucose during meals, decreases fasting blood glucose, improves glucose tolerance, and increases insulin sensitivity	(81)
AZ1	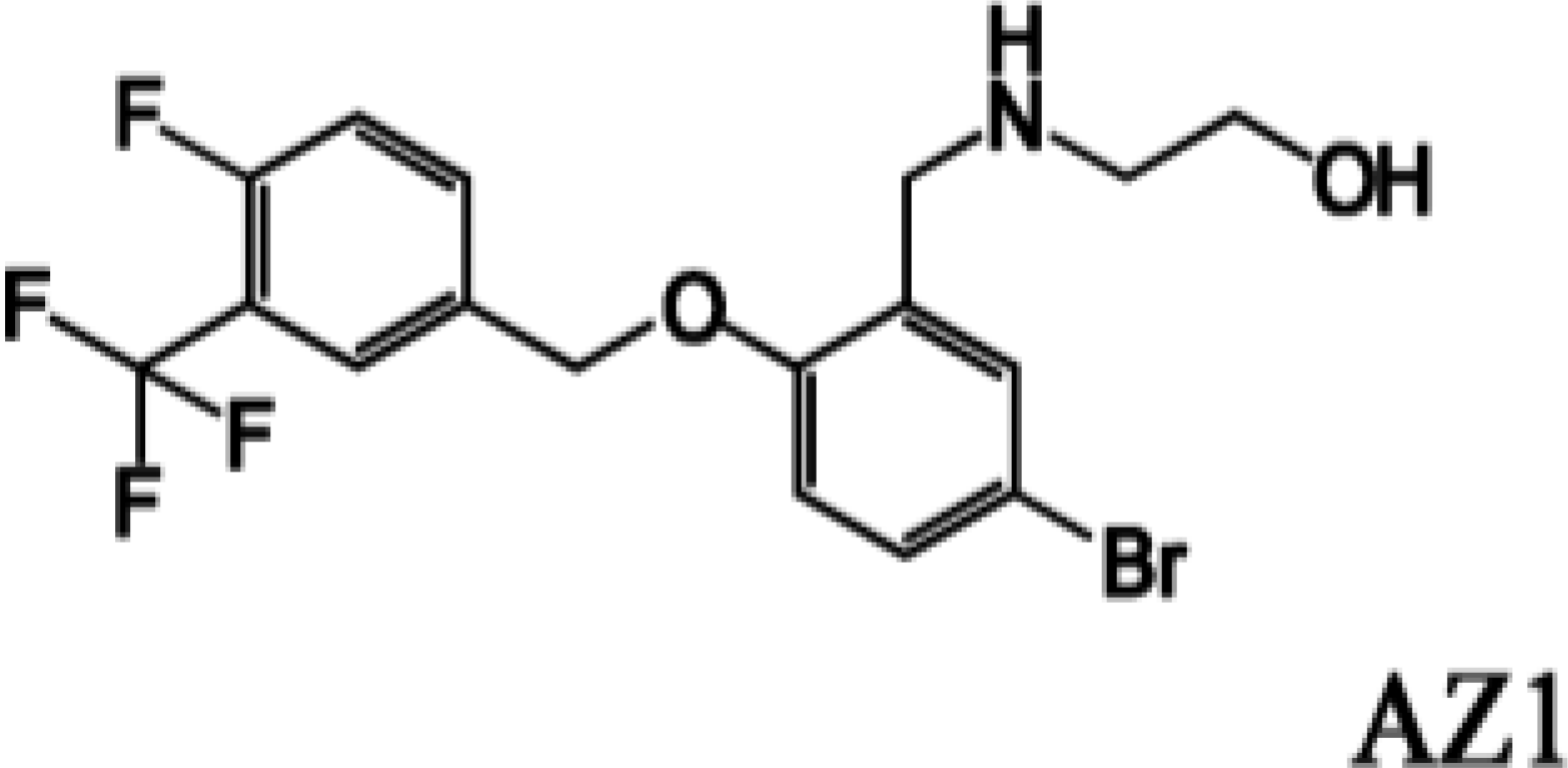	USP25	Aggravated diet-induced steatosis in mice	(55)

Among candidate molecules currently under investigation for diabetes treatment, MID-00935 has garnered significant attention as a novel MG53-IRS1 interaction disruptor. This compound specifically targets MG53, preventing its interaction with IRS1, thereby blocking MG53-mediated ubiquitination and degradation of IRS1. Ultimately, MID-00935 restores and enhances insulin signaling function. Animal studies have demonstrated that oral administration of MID-00935 significantly improves IR in diet-induced obese mouse models (33). Notably, phosphorylation at serine 255 of MG53 is a critical prerequisite for its E3 ubiquitin ligase activity. Studies have shown that when this site is mutated to alanine (MG53-S255A), the E3 ligase activity of MG53 is completely abolished, while its cytoprotective function remains intact. Further research demonstrated that this mutant confers protective effects against diabetic myocardial injury in db/db diabetic model mice. Moreover, MG53-S255A mutant mice constructed using gene-editing technology showed a phenotype of reduced myocardial injury, thereby genetically validating these findings (78). In summary, these studies collectively suggest that targeted inhibition of MG53 holds promise not only for improving systemic IR but also for providing novel intervention strategies for diabetes-related cardiac complications, demonstrating significant clinical translational potential.

In contrast, the USP14 inhibitor, IU1, demonstrates multi-target and multi-pathway intervention potential for the prevention and treatment of T2DM and its complications. In regulating hepatic glucose metabolism, IU1 effectively suppresses hepatic gluconeogenesis and reduces blood glucose levels by inhibiting USP14-mediated deubiquitination of the transcriptional coactivator CBP, thereby downregulating the expression of key gluconeogenic genes (64). In the context of neurological complications, studies have found that the natural product Sennoside A significantly improves cognitive dysfunction in a T2DM mouse model by inhibiting the USP14/ERK1/2 signaling pathway. Notably, IU1 similarly mimics this effect, further validating the critical role of this pathway in diabetic neuropathy (79). This series of studies establishes the importance of USP14 as a potential therapeutic target for diabetic neurological complications and suggests that the discovery of novel DUB inhibitors from natural products may represent a valuable approach for developing multi-target therapeutic strategies for diabetes in the future.

In addition to the aforementioned targets, the RPN11 inhibitor, Capzimin, has been demonstrated to ameliorate diabetes-related phenotypes in animal models. RPN11, also known as POH1 or PSMD14, belongs to the deubiquitinase family (80). Its primary function is to enhance the stability of target proteins by removing ubiquitin modifications, thereby participating in the regulation of various cellular processes. Recent studies demonstrated that intraperitoneal injection of capzimin significantly reduces postprandial and fasting blood glucose levels in db/db mice, a genetic model of diabetes, and effectively improves glucose tolerance and insulin sensitivity (81). These findings not only directly indicate the potential therapeutic value of Capzimin as an RPN11 inhibitor for T2DM intervention but also provide important experimental evidence for the development of novel diabetes treatment strategies targeting the proteasome-associated deubiquitinase RPN11. Therefore, targeting RPN11 and its mediated deubiquitination process represents a promising avenue for T2DM drug development.

However, not all deubiquitinase inhibitors yield the expected metabolic improvements. Suppression of certain targets may even trigger adverse metabolic responses. For example, in high-fat diet-induced mouse models of metabolic disorders, administration of the USP25-specific inhibitor AZ1 significantly increases triglyceride accumulation in the liver while suppressing fatty acid oxidation activity. Key signaling pathways regulating lipid metabolism, such as PPARα, also showed a weakening trend, leading to an overall aggravation of markers associated with hepatic steatosis (55). This paradoxical outcome suggests that in specific metabolic regulation contexts, inhibition of USP25 may compromise hepatic lipid homeostasis maintenance and even exacerbate metabolic imbalance. Therefore, in contrast to conventional inhibition strategies, moderately enhancing or restoring USP25 functional activity of USP25 may have therapeutic potential for improving lipid metabolism and alleviating hepatic steatosis. This study provides novel insights and directions for targeted interventions in metabolic diseases.

Targeting key components of the ubiquitination system has provided novel directions for mechanistic research and drug development in T2DM, offering opportunities to address traditionally “undruggable” targets and enabling tissue-specific interventions. However, this strategy faces multiple challenges because T2DM is a highly heterogeneous disease, and whether its complex pathological network can be remodeled by modulating a single E3 ligase or DUB requires systematic validation. Furthermore, most candidate molecules remain at the preclinical stage, and their *in vivo* specificity, long-term safety, and tissue selectivity require comprehensive evaluation. Therefore, future research should cautiously advance the translational application of these intervention strategies based on an in-depth analysis of disease heterogeneity.

## 5 Challenges, knowledge gaps, and future directions

Despite considerable progress in understanding the roles of E3 ubiquitin ligases and DUBs in T2DM, several important issues remain unresolved. First, most current studies are based on cell lines or animal models, and whether these findings can be extrapolated to human T2DM remains unclear (11). A few exceptions include the OTUD3 locus (rs78466831), which has been associated with T2DM risk in genetic studies. However, for the vast majority of enzymes discussed in this review, direct evidence from human cohorts or patient tissues is still lacking (82). Second, the same enzyme may exert opposite effects in different tissues or disease contexts. For example, TRIM32 promotes IR in the liver (34), induces β-cell death in the pancreas (44), but protects podocytes in diabetic nephropathy (48). This functional heterogeneity makes it difficult to predict the outcomes of systemic intervention (83). Third, many studies only report correlations between enzyme expression levels and metabolic phenotypes. Whether altered ubiquitination activity is a cause or a consequence of T2DM-related abnormalities remains unclear, and tissue-specific gain- or loss-of-function models are urgently needed.

Regarding experimental approaches, whole-body knockout animal models are widely used, but their limitations are increasingly recognized. Such models cannot distinguish tissue-specific functions of a given gene and may trigger compensatory changes that mask true phenotypes. Taking TRIM28 as an example, adipocyte-specific TRIM28 knockout mice exhibit the same obese phenotype as whole-body heterozygous knockout mice (84), whereas muscle-specific TRIM28 knockout does not cause significant systemic metabolic abnormalities (49). These results indicate that the effect of TRIM28 on increased body fat is primarily mediated by adipose tissue rather than skeletal muscle, and also suggest that other functionally redundant enzymes may compensate for the loss of TRIM28 in muscle tissue. This phenomenon is not uncommon in the ubiquitination system, given that the human genome encodes approximately 600 E3 ligases and 100 DUBs, making functional redundancy a general feature. Furthermore, the limitations of cell-based experiments should not be overlooked. Conventional cell culture conditions are relatively stable and cannot replicate the complex and dynamic metabolic environment of T2DM *in vivo*, including fluctuating glucose and lipid levels, dynamically changing inflammatory cytokines, and intricate hormonal regulatory networks. Therefore, findings obtained from cell lines often need to be further validated in animal models or even human samples to confirm their physiological relevance (85).

From the perspective of clinical translation, several practical challenges exist. First, the biological functions of ubiquitinating enzymes exhibit marked tissue dependence. Taking TRIM32 as an example, it is a harmful enzyme in the liver and pancreas but protective in the kidney. This functional heterogeneity makes systemic intervention extremely difficult. Therefore, when developing therapeutic strategies targeting TRIM32, simply using inhibitors may improve hepatic IR and β-cell survival, but could simultaneously impair its protective function in the kidney, leading to unexpected adverse consequences. Second, clinical translation also faces tool-related limitations. Commonly used animal models of T2DM (such as high-fat diet-induced models, db/db mice, and ob/ob mice) can mimic some features of human disease, but cannot fully recapitulate the heterogeneity and complexity of human diabetes. Moreover, there is currently a lack of circulating biomarkers that can reflect the activity of E3 ligases or deubiquitinating enzymes, making target engagement validation and dose selection in clinical trials difficult. The development of blood- or urine-based detection methods would facilitate the clinical translation of drugs targeting the ubiquitination system.

Future research should aim to make breakthroughs in the following areas. First, traditional whole-body knockout animals cannot distinguish tissue-specific functions of a given gene and are prone to compensatory changes that mask true phenotypes. Therefore, subsequent studies should employ conditional knockout or knock-in mice to achieve specific regulation of target enzymes in particular tissues (such as liver, adipose tissue, skeletal muscle, or pancreas), thereby elucidating their functions in the pathogenesis of T2DM. Second, single-level analysis is insufficient to fully reveal the complexity of the ubiquitination regulatory network. Future research should advance multi-omics integrative studies, combining single-cell transcriptomics, spatial transcriptomics, and ubiquitin proteomics to systematically investigate ubiquitination dynamics in T2DM-associated tissues across different cell types and metabolic states, and to quantitatively analyze ubiquitination modification profiles under various physiological or pathological conditions. Third, machine learning and artificial intelligence tools can be used to predict enzyme-substrate interactions, screen for highly selective small-molecule inhibitors or activators, and design novel drugs (such as PROTACs) that harness the ubiquitination machinery for therapeutic purposes, thereby accelerating the drug discovery process. Fourth, T2DM involves metabolic dysregulation across multiple organs, and single-target interventions often fail to achieve ideal effects. Simultaneously modulating two or more enzymes in different tissues may produce synergistic effects. For example, combining an MG53 inhibitor that improves skeletal muscle IR with a USP14 inhibitor that suppresses hepatic gluconeogenesis could intervene at different nodes simultaneously to enhance overall therapeutic efficacy. Of course, combination therapy would also require more systematic toxicological evaluation.

## 6 Short summary

This review highlights the central role of ubiquitination modifications in the pathogenesis of T2DM. From pancreatic β-cells, which are responsible for insulin synthesis and secretion, to insulin-sensitive peripheral tissues—including liver, skeletal muscle, and adipose tissue—and extending to target organs affected by diabetic complications such as blood vessels, heart, and kidneys, E3 ubiquitin ligases and DUBs collectively govern disease initiation and progression through tissue-specific precision regulatory networks. These findings position protein homeostasis regulation at the core of diabetes pathology research and provide a unified conceptual framework for understanding the systemic pathological network of diabetes from the novel perspective of “ubiquitination modification.” Nevertheless, significant challenges remain in this field. Most current findings remain at the preclinical validation stage, and their human relevance has yet to be confirmed. While numerous studies have reported statistical associations, direct causal evidence linking specific E3 ligases or DUBs to defined phenotypes remains limited. Ubiquitination regulation exhibits high tissue specificity, as the same molecule may exert diametrically opposite effects in different organs, making systemic drug administration potentially prone to unpredictable off-target effects. Future research should leverage tissue-specific gene manipulation technologies to validate the precise *in vivo* functions of key molecules while integrating proteomics with advanced interactomics approaches to systematically map comprehensive ubiquitination modification networks. Ultimately, translating insights from basic research into innovative therapies that precisely modulate ubiquitination homeostasis in specific tissues will open novel avenues for the targeted prevention and treatment of T2DM.

## Glossary

β-catenin: Cadherin associated protein beta 1
AKT: Protein kinase B
Cbl: Casitas B-lineage lymphoma proto-oncogene
CBP: CREB-binding protein
DDB2: Damage specific DNA binding protein 2
DDX3X: DEAD-box helicase 3 X-linked
dILPs: Drosophila insulin-like peptides
DUBs: Deubiquitinating enzymes
ERK1/2: Extracellular signal-regulated kinase ½
FASN: Fatty acid synthase
FABP: Fatty acid-binding protein
FBXW7: F-box and WD repeat domain containing 7
Fetuin-A: Alpha-2-HS-glycoprotein
FoxO1: Forkhead box O1
Gαs: G Protein subunit alpha s
GLUT4: Glucose transporter type 4
IBD: Inflammatory bowel disease
IDF: International Diabetes Federation
INSR: Insulin receptor
IR: Insulin resistance
IRS1: Insulin receptor substrate 1
IRE1α: Inositol-requiring enzyme 1 alpha
KMT2A: Lysine methyltransferase 2A
March5: Membrane associated ring-CH-type finger 5
MG53: Mitsugumin 53
MuRF1: Muscle RING Finger 1
NGN3: Neurogenin 3
Notch1: Neurogenic locus notch homolog protein 1
NRF2: Nuclear factor erythroid 2-related factor 2
OTUB1: The deubiquitinase OTU domain-containing ubiquitin aldehyde binding protein 1
OTUD3: OTU domain-containing protein 3
OTUD5: OTU domain-containing protein 5
OTUD1: OTU domain-containing protein 1
PPARα: Peroxisome proliferator activated receptor alpha
PEPCK1: Phosphoenolpyruvate carboxykinase 1
Perilipins: Lipid droplet coat proteins
PKG2: Protein kinase, cGMP-dependent, type II
PPARδ: Peroxisome proliferator-activated receptor delta
PPARγ: Peroxisome proliferator-activated receptor gamma
PTEN: Phosphatase and tensin homolog
RIPK1: Receptor interacting serine/threonine kinase 1
RNF167: Ring finger protein 167
RNF186: Ring finger protein 186
RPN11: Regulatory particle non-ATPase 11 (POH1/PSMD14)
SMAD7: SMAD family member 7
SMURF1: SMAD specific E3 ubiquitin protein ligase 1
Stat3: Signal transducer and activator of transcription 3
TAK1: TGF-beta activated kinase 1
T2DM: Type 2 diabetes mellitus
TRAF6: TNF receptor associated factor 6
TRIM21: Tripartite Motif Containing 21
TRIM32: Tripartite Motif Containing 32
TRIM28: Tripartite motif containing 28
UCHL1: Ubiquitin C-terminal hydrolase L1
USP2: Ubiquitin-specific protease 2
USP5: Ubiquitin-specific protease 5
USP7: Ubiquitin-specific protease 7
USP10: Ubiquitin-specific protease 10
USP11: Ubiquitin-specific protease 11
USP14: Ubiquitin-specific protease 14
USP15: Ubiquitin-specific protease 15
USP18: Ubiquitin-specific protease 18
USP25: Ubiquitin-specific protease 25
USP30: Ubiquitin-specific protease 30
VDAC2: voltage-dependent anion-selective channel 2
WWP2: WW domain containing E3 ubiquitin protein ligase 2
YWHAB: the tyrosine 3-monooxygenase/tryptophan 5-monooxygenase activation protein β
